# Predicting the mortality due to Covid-19 by the next month for Italy, Iran and South Korea; a simulation study 

**Published:** 2020

**Authors:** Sajad Shojaee, Mohamad Amin Pourhoseingholi, Sara Ashtari, Amir Vahedian-Azimi, Hamid Asadzadeh-Aghdaei, Mohammad Reza Zali

**Affiliations:** 1 *Gastroenterology and Liver Diseases Research Center, Research Institute for Gastroenterology and Liver Diseases, Shahid Beheshti University of Medical Sciences, Tehran, Iran *; 2 *Basic and Molecular Epidemiology of Gastrointestinal Disorders Research Center, Research Institute for Gastroenterology and Liver Diseases, Shahid Beheshti University of Medical Sciences, Tehran, Iran *; 3 *Trauma Research Center, Nursing Faculty, Baqiyatallah University of Medical Sciences, Tehran, Iran *

**Keywords:** COVID-19, Coronavirus, Mortality, Iran, Italy, South Korea

## Abstract

**Aim:**

To estimate the number of confirmed cases and the rate of death and also to investigate the cause of death in Italy, Iran and South Korea in the next month.

**Background:**

Growing number of confirmed and deaths cases from the coronavirus worldwide, particularly in Italy, Iran and South Korea, has resulted concerns about the future of these countries and their deterioration. Also the European region is likely to face more casualties due to the delay in the virus reaching most of its regions and, of course, as the trend continues.

**Methods:**

We conducted a simulation in both current and ideal situation for the next month to predict the death rate and examine the reason for the difference in Italy, Iran and South Korea individually. If we assume the cultural and political factors and age pyramids distribution are similar across regions, the differences are mainly due either to the heavier health-care burden owing to the larger population or to the medical facilities diversities.

**Results:**

Our results for Italy showed higher death number, but the rate would be more for Iran. South Korea is also expected to have a smaller increase in the number of confirmed cases and deaths compared to Iran and Italy by the next month.

**Conclusion:**

Given the prevailing conditions around the world and the increasing number of casualties, it is essential that all countries, especially those with fewer days of involvement, shall do their best to avoid major losses and damages.

## Introduction

 To date, the number of countries affected by the coronavirus has reached more than 100, and as the virus progresses it will likely become epidemic. Given the increasing outbreak of infected cases and deaths worldwide, the fears and anxieties that arise are challenging (1,2). As increased of humanity's awareness and knowledge and employing of calibrated interventions during the course of the outbreak, fatality estimates would be necessary for policy makers (3).

Given the growing importance of the issue, the World Health Organization has collected and disseminated a wealth of coronavirus information on confirmed, recovered, and death cases. The information is available on RamiKrispin dataset (4) as of January 22, 2020 and is updated daily. At a glance, people whom as new confirmed or recovered or death cases are entered daily, forming a queue system (5). In fact, every new confirmed person who comes in daily, after a while, either recovers or dies.

With the exception of China, where the outbreak began, Italy, Iran and South Korea were the countries with the highest number of confirmed cases and deaths with far differences from the other countries (especially in Italy and Iran, up to March 15) and also had the highest number of days of involvement with the virus compared to most countries. The differences in the number of deaths could be due to the large number of confirmations that could be considered an indirect indicator of the heavier health-care burden on these countries (6). This subject proposed by the study of Yu et al. (7) on the unevenness of the distribution of high-quality health-care resources in different provinces and cities of China.

**Table 1:** Outbreak and death rate of Coronavirus in Italy, Iran and South Korea from 15 March to 15 April

Country	Date	Confirmed Cases^*^	Death Cases^*^	Death Rate^**^
Italy	1/31/2020 to 3/15/2020	24747	1809	7.31
	Next 10-day forecast	61725(357)	4840(99)	7.84(0.17)
	Next 20-day forecast	114803(865)	9249(240)	8.06(0.22)
	Next 30-day forecast	183979(1580)	18395(437)	8.18(0.25)
South Korea	1/22/2020 to 3/15/2020	8162	75	0.92
	Next 10-day forecast	11419(98)	130(13)	1.14(0.11)
	Next 20-day forecast	14807(221)	199(29)	1.34(0.20)
	Next 30-day forecast	18327(379)	283(51)	1.55(0.28)
Iran	2/19/2020 to 3/15/2020	13938	724	5.19
	Next 10-day forecast	28788(303)	2215(91)	7.70(0.32)
	Next 20-day forecast	48226(823)	4562(255)	9.46(0.54)
	Next 30-day forecast	72251(1605)	7770(502)	10.76(0.72)

* The numbers are written as sum or mean (sd); ** Rates are expressed as percentage

The main purpose of the current study was to estimate the number of confirmed cases and the rate of death and also to investigate the cause of death in the most prevalent countries in the next month. Accordingly, we conducted a simulation study to predict the possibly future situation using the current situation in Italy, Iran and South Korea. 

## Methods

The number of inputs or successive inputs over a period of time constitutes a sequence of random variables called the input process (5). The input values have a Poisson distribution with the specified parameter p. Given the number of recent days of virus Outbreak onset, the p-parameter is estimated by countries and cases, using a simple linear regression model (8). These regression models makes it possible to predict p estimates which is the slope of the regression lines, in the upcoming days.

We simulated confirmed and deaths data using input process, individually for Italy, Iran and South Korea in a current scenario based on the second half the number of days of their conflict. Analyzes were performed from March 15 to April 15 by applying R version 3.6.1 software on RamiKrispin dataset (4). An ideal scenario also employed to predict the number of confirmed cases based on a decreasing pattern in China.

We have arranged the results for the next 10, 20 and 30 days by current scenario in individually countries with 10000 replications in Table 1. 

## Results and Discussion

South Korea, Italy and Iran reported the first positive cases of Coronavirus on January 22, January 31 and February 19 respectively, and to date (March 15) in all three countries, the number of cases according to WHO statistics have catastrophic increased. Our results showed the number of cases infected to the coronavirus in Italy is higher than in South Korea and Iran by next month (183,979 Italian vs. 18,327 South Korean and 72,251 Iranian), while South Korea has the lowest mortality rate (1.55 vs. 8.18 and 10.76 in Italy and Iran) (Table 1). At the Iran national level, with the exception of Tehran and a few other cities, care facilities are low (9,10) and due to the outbreak of the virus in almost whole of this country, the possibility of deaths in the cities with low facilities is high. Although, Definitions identifying positive cases of the virus may also vary across countries, as well as countries maybe went underreported in counting of confirmed and death cases.

The increase in the number of patients in European countries indicates that the region has become more critical in recent days (11), and as the outbreak onset has been to delay further than South Korea, Italy and Iran; the same fate would be expected if the virus is not seriously tackled.

Our results have been in line with the trend of virus spread in these three countries in recent days, which may, however, decreases in the number of cases in the coming days with more safety observance for individuals in each community and providing an appropriate foundation for preventing the spread of the virus by health centers. In addition, by remaining of our simulation results proved if Iran, Italy and South Korea were able to restrain the virus by preventive measures, thereby reducing the upward slope and even initiating its downward slope, by actually implementing a reduced proportion of cases in China from the last 30 days of the first infected reported till today, the number of confirmed cases in Italy after 10, 20, and 30 days will reach to 28,925, 33,281 and 37,661, in Iran to 15,344, 16,813 and 18,288 and in South Korea to 8,250, 8,342 and 8,434 respectively. Making such situation looks ideal and requiring sufficient financial and human resources, safeguarding medical personnel and staff as front line against the Coronavirus, and ultimately concentrating all national activities on recovering society to get rid of the virus.

## Conflict of interests

The authors declare that they have no conflict of interest.
